# Differential indexing in Kamang: a viewpoint alternation

**DOI:** 10.1515/lingvan-2023-0052

**Published:** 2024-01-01

**Authors:** Katherine Walker

**Affiliations:** 168063 Amsterdam Center for Language and Communication, University of Amsterdam, Amsterdam, The Netherlands

**Keywords:** Alor-Pantar, alternation, differential argument indexing, split-S, agreement

## Abstract

In Kamang (Alor-Pantar, Indonesia), some verbs alternate between indexing the S or P argument with a prefix (from several different series) and occurring unprefixed; that is, Kamang has differential argument indexing. Through a qualitative study of a spoken-language corpus, this paper investigates the alternation between one of the prefix series and zero-marking. Previously described as indicating increased patientivity on intransitive motion and posture verbs, the alternation is here analysed in terms of a shift in event view: unprefixed verbs express events holistically, while prefixed verbs shift the viewpoint towards the “elaboration phase”, the temporal and causal middle and end of an event. Elaboration constructions are associated with resultative and completive semantics and, relatedly, greater affectedness of the undergoer argument. Cross-linguistically, such constructions (e.g., resultatives and middles) are frequently subject to lexical restrictions, and the same applies to the Kamang alternation.

## 1 Introduction

Differential argument indexing (DAI) is a type of alternation whereby arguments are expressed differently on different verbs or in different contexts.1Indexing (verbal clitics or affixes) contrasts with flagging (case marking, adpositions); differential argument marking includes both (Witzlack-Makarevich and Seržant 2018). Other terms that overlap with DAI as defined here include differential agreement (Lazard 2005), differential argument realization (Kratochvíl 2014), and sporadic agreement (Corbett 2006: 17; Fedden 2019). For differential object indexing, see Iemmolo (2011). For instance, in Kamang (Alor-Pantar, Indonesia), *tak* ‘run’ has no index for the S argument in (1a) and a third person index *ge-* in (1b).2In the text, S indicates the single argument of an intransitive verb; P, the more patient-like argument of a transitive verb; and A, the more agent-like argument of a transitive verb. Examples follow the Leipzig Glossing Rules. Abbreviations used: 1/2/3 first/second/third person; /a/, /e/, /o/ prefix series labels; A actor; agt agent; an animate; cmn common person (reciprocal, generic); cmp complement; dem demonstrative; dir directional marker; excl exclusive; incl inclusive; ipfv imperfective; lnk linker; pfv perfective; pl plural; purp purposive; rel relativizer; sg singular; spec specific; take semi-verb in complex predicates; U undergoer. Kamang examples from fieldnotes provided by Antoinette Schapper and George Saad differentiate between elicited (stand-alone phrases from context-free elicitation) and spontaneous speech extracted from a wider context. Citations in square brackets indicate texts in the annotated sub-corpus (Schapper et al. forthcoming), all classed as spontaneous. Glosses from published sources are largely retained, except for Kamang prefixes, which are glossed with their series name /a/, /e/, or /o/. English examples are based on author intuition if no citation is given; some English translations from the corpus have been lightly edited for clarity.

(1)

a.

**Table:** 

*kui*	*tak*
dog	run
‘The dog runs.’	
(Schapper 2014: 326)

b.

**Table:** 

*kui*	*ge-tak*
dog	3./e/-run
‘The dog ran off (was forced to run).’	
(Schapper 2014: 326)

Previously, differential prefixes like those in (1b) have been described as indicating greater patientivity on intransitive motion and posture verbs (Schapper 2014: 325–326).3The alternants are therefore not semantically equivalent (cf. Goldberg 1995): of typological interest is precisely the range of semantic differences that the same kind of formal alternation – DAI – can express. Based on the qualitative analysis of a small corpus of spoken Kamang, the present study expands this analysis. It shows that the alternation is less restricted than previously described: it applies to verbs from other semantic classes and to both intransitive and transitive verbs, though lexical restrictions persist. Further, while patientivity remains a factor, I present a new analysis in terms of a shift in event view, which is otherwise morphologically unmarked. That is, unprefixed verbs like in (1a) express events holistically, while prefixed verbs like in (1b) shift the viewpoint towards what I call the “elaboration phase”, the temporal and causal middle and end of an event. Elaboration constructions are associated with resultative and completive semantics and, relatedly, greater affectedness of the undergoer argument. Cross-linguistically, elaboration-phase constructions (e.g., resultatives and middles) are also frequently subject to lexical restrictions. The event view and features of the elaboration phase are described in Section 2. Section 3 presents the Kamang language and data, and Section 4 summarizes the findings.

## 2 The event view and viewpoint shift

In Croft’s (1994) event view, an event has three phases: cause → become → state. Both causal and temporal chains are evoked by the left-to-right arrows: from actor to undergoer, and from initiation to completion (DeLancey 1982; Fauconnier 2013). Each phase includes the phases to the right of it. Hence, a construction may encode all three phases (“causative”, e.g., *the rock broke the window*), the two phases become and state (“inchoative”; *the window broke*) or the state phase only (“stative”; *the window is broken*; Croft 1994: 93). Causatives are generally transitive and include both A and P arguments, while inchoatives and statives are generally intransitive; however, any phase can be realized as an intransitive with an S argument (Croft 1994: 109).

Languages provide various means for encoding different phases and for shifting or narrowing the viewpoint. Common grammatical systems for this purpose are voice and diathesis: the categories that align with each viewpoint are given in Table 1. For instance, voice alternation in English shifts from the standard “causative” viewpoint encompassing all three phases in an active clause – *the rock broke the window* – to focus on the middle and end phases in a passive construction: *the window was broken (by the rock)*. In this alternation, the cause phase is semantically greyed out, which is realized syntactically by encoding the actor in a non-obligatory prepositional phrase. From a holistic view of the whole event, the passive shifts focus to the end of the causal chain, the undergoer, which becomes the subject.

**Table 1:** Event phases and associated categories (after Croft 1994: 113).

Event	Causative	Inchoative	Stative
Event phases	cause → become → state	become → state	state
Argument role	A/S	P/S	P/S
Semantic role	Actor	Undergoer	Undergoer
Voice	Active	Middle	Passive
Diathesis	Causative	Inchoative	Resultative

Though the event view is tripartite, binary alternations are frequent. DAI systems of S arguments (split-S, semantic alignment; see, e.g., Wichmann 2008) are one example, which Croft aligns with the event view as shown in (2). That is, “active” intransitive predicates align under cause (i.e., include all three event phases), while “stative” predicates align under become and state. However, many languages, such as Dobel (Austronesian, Indonesia), group become with cause rather than with state, called a dynamic-stative split (Klamer 2008: 248). In Dobel, patientive postclitics index S on stative predicates (e.g., *ŋeŋan=ni* [heavy=3sg.U.an] ‘he is heavy’; Hughes 2000: 148), while agentive proclitics index S on almost all other intransitive predicates. This includes those without initiators, such as inchoative *ŋ=kwoy* [2sg.A=die] ‘you die’ (Hughes 2000: 137). Given the potential confusion between “active” and “dynamic”, Croft’s split (cause vs. become-state) will be referred to here as an initiation-elaboration split.

(2)

**Table:** 

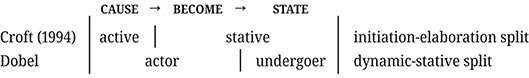

An initiation-elaboration split is described for Pilagá (Guaicuruan,4Naming convention from Glottolog (Hammarström et al. 2022); this family is also known as Guaykuruan (Vidal 2008). South America), which indexes S/A arguments with either “set A” or “set B” affixes (Vidal 2008).5A separate paradigm of “object markers” indexes P arguments. For some verbs, affix alternation is claimed to express a viewpoint split: events viewed from the start (causative) encode S/A with set A affixes, while events viewed at the moment of change (inchoative) or their endpoint (resultant state) encode S/A with set B affixes (Vidal 2008: 429). In (3), the set A prefix indexes the actor of an event viewed from the start, while a set B prefix indexes an undergoer S argument of an event with no cause phase. Set B affixes are also associated with greater affectedness (Vidal 2008: 430); this is a common property of an endpoint view, as detailed below.6In the gloss of (3b), Val1 (“valency derivation 1”) “does not change the verb valency”, but may be “related to affectedness” (Vidal 2001: 173–174).

(3) Pilagá

a.

**Table:** 

*ya-čemat-iyi*
setA.3-roast-dir
‘They roast (fish).’
(Vidal 2001: 174)

b.

**Table:** 

*na-čemat-aʕak*
setB.3-roast-Val1
‘It is roasted.’
(Vidal 2001: 174)

Constructions that encode or shift focus to the elaboration phase are associated with a number of properties, of which three are relevant here. First, they may express telic or atelic aspect (“change of state” and/or “unbounded processes and activities”, respectively; Arkadiev 2008: 105–106), since the elaboration phase encompasses both inchoatives (change of state: telic) and statives (atelic). For instance, resultatives “express a state implying a previous event” (Nedjalkov and Jaxontov 1988: 6); that is, that a change of state has (just) taken place.

Another aspectual category associated with the elaboration phase is the completive: “to do something thoroughly and to completion” (Bybee et al. 1994: 18). Fauconnier (2013) links completive aspect to endpoint focus, which entails centring the undergoer and thus creating a stronger association with properties of a prototypical transitive P argument, such as greater affectedness or low volitionality (Fauconnier 2013; cf. Hopper and Thompson 1980). This is the second property: the association between the undergoer argument in an elaboration alternant and features of a prototypical P argument.

Third, initiation-elaboration alternations are often subject to lexical restrictions and lexicalization. That is, only some verbal bases alternate and alternants may have idiosyncratic meanings. This is attested, for instance, in cross-linguistic investigations of resultative and middle constructions (Beavers 2012; Kemmer 1994; Nedjalkov and Jaxontov 1988). For verbal bases that do alternate, the same semantic classes recur in the literature. These include translational motion verbs, illustrated in (4), body action or body posture verbs, in (5), and emotion/cognition predicates, in (6) (see Bybee et al. 1994; Kemmer 1993: 147; Nedjalkov and Jaxontov 1988; Sil’nickij 1988). Note the lexical restrictions illustrated in (5), where semantically similar verbs *sich hinsetzen* ‘sit down’ and *aufstehen* ‘stand up’ admit different construction types, and in (6), where these verbs do not alternate with an unaffixed variant.

(4)

a.

**Table:** 

Spanish (Indo-European)	
Middle	*ir-se* ‘go away’, *caer-se* ‘fall’
Active	*ir* ‘go’, *caer* ‘fall’
(Kemmer 1994: 186)

b.

**Table:** 

English (Indo-European)	
Resultative	*he is gone*
Active	*he went*
(cf. Bybee et al. 1994: 54)

(5)

**Table:** 

German (Indo-European)	
Middle	*sich hinsetzen* ‘sit down’, *sich hinlegen* ‘lie down’
Active	(*sich) *aufstehen* ‘stand up’
(Kemmer 1994: 185)

(6)

**Table:** 

Fang (Bantu): middle constructions with *-a* (“associative”, see also Dom et al. 2016)
*-yá-á* ‘to get angry’ (*-yá)
*-zob-a* ‘to regret; to be embarrassed by unpleasant news, be sad’ (*-zob)
*-sím-á* ‘to remember, think’ (*-sím)
(Bostoen and Nzang-Bie 2010: 1279)

Emotion/cognition predicates illustrate that the argument has more features of a prototypical P: arguments of such predicates “naturally exhibit low control, will, or instigation” (Vidal 2008: 428). They are also a common locus for differential S/A marking cross-linguistically (Nichols 2008: 129). Also, in Pilagá, there is pervasive lexicalization in mental state verbs (Vidal 2008: 430). Regardless of semantic class, alternation frequently results in lexicalized – that is, idiosyncratic – meaning change. For example, in Russian the resultative form *rassejan* ‘scattered’ has an unpredictable lexicalized meaning ‘absent-minded’ (Nedjalkov and Jaxontov 1988: 61).

These three properties of elaboration-view constructions – mixed telicity, greater affectedness, and lexical restrictions or lexicalization – are shown below to characterize the Kamang /e/-series alternation. I therefore analyse it as an initiation-elaboration split, which nevertheless differs from the Pilagá split in important ways.

## 3 Case study: Kamang /e/-series

### 3.1 Language and data

Kamang is a severely endangered Alor-Pantar (Papuan/non-Austronesian) language spoken by around 6,000 people in Indonesia (Schapper 2014: 286–287). The language possesses up to seven prefix paradigms that index the S or P argument. Table 2 gives three of the most common prefix paradigms – the /a/-series, /o/-series, and /e/-series7
Schapper (2014) used the labels “patientive”, “locative”, and “genitive” for these prefixes. – plus the dative and self-benefactive paradigms, which are phonologically similar to the /e/-series.

**Table 2:** Selected Kamang prefix paradigms (Schapper 2014: 322, 331).

	/a/-series	/o/-series	/e/-series	Dative	Self-benefactive
1sg	*na-*	*no-*	*ne-*	*nee-*	*ne′-*
2sg	*a-*	*o-*	*e-*	*ee-*	*e′-*
3	*ga-*	*wo-*	*ge-*	*gee-*	*ge′-*
cmn ^a^	*ta-*	*to-*	*te-*	*tee-*	*te′-*
1pl.excl	*ni-*	*nio-*	*ni-*	*nii-*	*ni′-*
1pl.incl	*si-*	*sio-*	*si-*	*sii-*	*si′-*
2pl	*i-*	*io-*	*i-*	*ii-*	*i′-*

^a^The “common” prefixes are used for reciprocal and generic reference (Schapper 2014: 314).

Around a third of verbal bases obligatorily bear an index from a single prefix series; the rest are either never prefixed, or participate in DAI. Various alternation types are attested – between prefixation and zero-marking, or between prefixation with indexes from different series (see Fedden et al. 2013, 2014; Schapper 2014; Walker et al. 2023). This study focuses on the alternation presented in (1) above and in (7) below; namely, between /e/-series prefixation and zero-marking.

(7)

a.

**Table:** 

*dum=a*	*nih*
child=spec	sit
‘The child sits.’	
(Schapper 2014: 326)

b.

**Table:** 

*dum=a*	*ge-nih*
child=spec	3./e/-sit
‘The child sat/lived (there for a long time).’	
(Schapper 2014: 326)

The /e/-series alternation has been described as indicating greater patientivity of S on motion and posture verbs (Schapper 2014). This has two typical interpretations: greater affectedness and/or greater duration. In (1a), zero-marking is used for a less-affected S argument whereas, in (1b), an /e/-series prefix “indicates the dog’s running away was caused by an external event, such as someone kicking at the dog” (more affected; Schapper 2014: 255). In terms of duration, the prefixed form indicates that a motion or posture was held for a long time. In (7b), *nih* ‘sit’ acquires the “somewhat lexicalized meaning … ‘live, reside’” (Schapper 2014: 326) with an /e/-series prefix.

The present study aims to provide a more detailed picture of the /e/-series alternation through a qualitative analysis of examples attested in a corpus of spoken Kamang. The corpus includes monologic narratives, conversations, and structured (i.e., using stimuli) and unstructured elicitation recorded by Antoinette Schapper and George Saad between 2010 and 2020.8Some elicitation involved one speaker judging the acceptability of certain verbs with /e/-series prefixes and zero-marking and describing the meaning change. Meaning differences elicited in this way are indicated in the description of the relevant examples. Part of this corpus – annotated for grammatical relations and animacy with the GRAID schema (Haig and Schnell 2014, 2022) – is publicly available (Schapper et al. forthcoming). Target verbal bases were those recorded in the lexicon or the annotated sub-corpus as alternating between zero-marking and /e/-series prefixation. Utterances containing these verbs were then extracted for comparison.

There are two points of note. First, plural forms of the /e/-series are homophonous with /a/-series plurals, which may lead to ambiguity. To confirm alternation with the /e/-series, verbal bases needed to be attested at least once with an unambiguous singular prefix (all of them were). Second, the /e/-series is phonologically similar to the dative and self-benefactive series. The phonetic differences between the three series in natural speech can be minimal if not imperceptible (Al-Gariri 2022). Since the dative prefix occurs only very rarely (Fedden and Brown 2017: 424–426; Schapper 2014: 324), ambiguity with the /e/-series is unlikely. However, the self-benefactive is frequent in discourse. To minimize the chance of including self-benefactives, I selected examples that (i) previous researchers had glossed as /e/-series and (ii) had accessible recordings in which no glottal stop was audible. Some examples found only in elicitation data could be discarded when the speaker explicitly mentioned the glottal stop (by referring to the glottal stop grapheme <′>). Consequently, only the clearest instances of /e/-series prefixation were included in the study, totalling 123 tokens for 21 lexical verbs. Some verbs occur much more frequently with zero: here, 20 examples were selected for comparison, prioritizing good quality recordings and clear surrounding context. Given the modest sample size (a common challenge in research on endangered languages) and the exclusion of unclear cases, future research will likely find that additional verbal bases can alternate.

Several features of Kamang grammar are relevant to this study (for a full description, see Schapper 2014). There are several ways to mark aspect: non-obligatory perfective and imperfective suffixes, stem suppletion, and aspectual serialization constructions. Aspectual serialization involves a sequence of two verbs, the second of which expresses aspect. Completive aspect is marked by *lai* ‘finish’ and continuous aspect by *dii* ‘lie’. The latter is illustrated in (8).

(8)

**Table:** 

*an*	*we*	*dii*	*me*	*dii=a*
thus	go	lie	come	lie=spec
‘(they) continued to come and go like this …’				
(Schapper fieldnotes, spontaneous)				

Also relevant here is the resultative construction: “one verb encodes the result of the event denoted by another verb. Result verbs are intransitive and occur as the final verb in the serialization” (Schapper 2014: 345). As shown in (9), the resultant state of the S argument is expressed when the previous verb is intransitive (‘fall down’).

(9)

**Table:** 

*mooi*	*bong*	*nok*	*kok*	*silang*	*dii*
banana	tree	one	fall.down	descend	lie
‘A banana tree came falling down.’					
(Schapper 2014: 345)					

If the previous verb is transitive, the final verb expresses the resultant state or position of P (Schapper 2014: 346). In (10), the P argument (semantically a location) of *tal* ‘make fence’ is *kadii* ‘house’, which in turn is the S argument of the resultant state *beela* ‘surround(ed)’.

(10)

**Table:** 

*Lukas*	*kadii*	*go-tal*	*beela*
Lukas	house	3./o/-make.fence	surround
‘Lukas puts up a fence around the house.’			
(Schapper 2014: 346)			

In other verb sequences, the final verb expresses a property of the A argument of the preceding transitive verb. While the final verb in the resultative construction in (10) expresses a property of P, the final verb in an otherwise identical construction in (11) expresses a property of A. That is, ‘a man’ is standing, not the child he is holding.9This utterance was elicited based on a video clip (Fedden et al. 2010a) in which the child is held in the air at an angle; ‘stand’ can only refer to the man holding her. There is currently no evidence that prefixation behaviour differs according to whether the final verb expresses the resultant state of the S, A, or P of the previous verb, but this remains a question for future research.

(11)

**Table:** 

*lami*	*saak*	*nok*	*ge-dum*	*ga-buh*	*latsi*
male	old	one	3./e/-child	3./a/-cradle	stand
‘A man stands holding his child.’^10^					
(Schapper fieldnotes, elicited)					

10 /e/- and /a/-series prefixes also index possessors on alienably and inalienably possessed nouns, respectively; see Schapper (2014: 311–313).

Finally, Kamang does not require any arguments in the clause to be overt (e.g., in (8) the S argument ‘they’ is unexpressed). Independent arguments (pronouns or lexical noun phrases) are permitted to co-occur in a clause with the prefix that indexes them, although co-occurrence is generally dispreferred (see Walker et al. 2023).

### 3.2 Kamang /e/-series alternation

Verbal bases that participate in the /e/-series alternation are listed in Table 3 along with their frequencies in each construction. The translation is of the unprefixed lexical base; where prefixation leads to an idiosyncratic meaning change – a common feature of elaboration constructions – this is given in the rightmost column. Note that unprefixed posture verbs can have both telic ‘stand up, sit down’ and atelic readings ‘stand, sit’ (cf. Schapper 2014: 326).

**Table 3:** Verbal bases exhibiting differential /e/-series indexing. Parentheses indicate alternations with scant data; “>20” indicates that 20 examples were selected from a larger total (see Section 3.1).

	Unprefixed		Prefixed with /e/-series	
Verb base	Meaning	Count	Idiosyncratic meaning	Count
Posture verbs				

*nih*	sit (down)	>20	live; reside	7
*dii*	lie (down)	>20	–	5
*latsi*	stand (up)	>20	–	4
*(laka)*	hang (up)	n/a^a^	be naked	n/a

Motion verbs				

*sila/silang*	descend	>20	–	17
*te*	go up straight	19	–	7
*yaangme (yaangma)*	come down (pfv)	16	–	1
*yaa*	go; reach	>20	go home	22
*tak*	run	20	run away	12

Other verbs				

*awila′*	fill (intr.)	4	–	1
*fanee*	work	15	strike	17
*paisan/paisang*	be bright; shine	2	explanation/explain	2
*(fun)*	touch	5	contribution/contribute	1
*taa*	sleep	>20	–	1
*beta*	bump into; barge	2	–	3
*baila′*	buy	3	–	2
*balkei*	sell	2	–	1
*sika*	finish	3	–	4
*mitan*	complete	7	understand	1

(Emotion/cognition verbs)				

*(maringki)*	startle(d)	1	–	3
*(biee)*	afraid	1	–	9
*(mai)*	hear	3	–	3

Total		223		123

^a^No clear example of alternation was available for the present study; alternation of *laka* is reported by Fedden and colleagues (2014: 63).

The items in parentheses in Table 3 were discounted due to insufficient data in the corpus to fully substantiate the alternation (e.g., due to difficulties in ruling out a transitivity difference). Three of these are emotion/cognition verbs (*maringki* ‘startle’, *biee* ‘afraid’, and *mai* ‘hear’): as mentioned, cross-linguistically such verbs frequently participate in DAI of S, and in elaboration constructions like resultatives and middles. Also relevant, then, is the idiosyncratic meaning shift from *mitan* ‘complete’ to the emotion/cognition verb ‘understand’ when prefixed.

Aside from emotion/cognition verbs, Table 3 shows the groups of motion and posture verbs, which Schapper (2014) previously identified as participating in /e/-series alternation. These semantic groups are also common in elaboration constructions; nevertheless, there is evidence of lexical restrictions even within a semantic class. For motion verbs, *tak* ‘run’ alternates, but semantically similar verbal bases like *maa* ‘walk’, *loo* ‘walk’, and *lila* ‘fly’ are not (yet) attested with /e/-series prefixes. For posture verbs, lexicalization is manifested in the idiosyncratic meaning difference between unprefixed *nih* ‘sit’ and ‘live; reside’ when prefixed. The final group in Table 3 is labelled “other” due to its heterogeneity. It includes verbal bases that seem inherently intransitive and non-volitional (‘sleep’) and inherently transitive and volitional (‘buy’, ‘sell’), as well as bases that appear to be inherently durative (‘work’, ‘be bright; shine’) or inherently punctual (‘bump into’, ‘finish’).

In terms of systematic meaning differences between the alternants, increased duration and affectedness is only part of the story. In (12a), the S argument has been in position for a long time, yet *latsi* ‘stand’ is zero-marked. Conversely, in the prefixed *ge-latsi* in (12b), the S argument goes volitionally to stand ‘up on top’ and does not remain there long. It is difficult to impute a “greater affectedness” reading here.

(12)

a.

**Table:** 

*waima*	*ang*	*tanaa*	*ge-kaang*	*tung*	*mane*	*mi*	*latsi-ma*
drum	dem	still	3./e/-be.present	up.there.straight	village	in	stand-pfv
‘the drum is still present, stood up there in the village’							
(Schapper fieldnotes, spontaneous)

b.

**Table:** 

*yaa=nte*	*woi*	*taa*	*ge-latsi*
go=but	stone	top	3./e/-stand
‘… and (he) went and stood up on top’			
[kamang_frog]

While greater affectedness is often a property of arguments indexed with the /e/-series, it is one of several properties associated with elaboration constructions, including completion, inchoativity, resultativity, and features of middles. As the following examples illustrate, the /e/-series alternation is best analysed as a viewpoint split between zero-marked verbs that express events holistically – either with all three phases, or without internal phasal partitions – and /e/-series-marked verbs that shift focus to the elaboration phase.

Example (13) shows two almost identical resultative serializations, the final verb *dii* ‘lie’ expressing the position of the P argument of the preceding verb *kuh* ‘cover’: *botil* ‘bottle’ in (13a) and *sukuu* ‘hole’ in (13b).

(13)

a.

**Table:** 

*botil*	*ge-tangpi*	*me*	*botil*	*wo-kuh*	*dii*
bottle	3./e/-cap	take	bottle	3./o/-cover	lie
‘The lid closes the bottle.’					
(Schapper fieldnotes, elicited)

b.

**Table:** 

*woi*	*baai*	*nok*	*me*	*see*	*sukuu*	*ang*	*wo-kuh*	*ge-dii*
stone	great	one	take	arrive.ipfv	hole	dem	3./o/-cover	3./e/-lie
‘(She) took the big stone and blocked the hole.’								
[kamang_aribei]

Unprefixed *dii* in (13a) is best analysed as a state proper with no internal phases (the utterance was elicited to describe a static picture). In (13b), the utterance is part of a narrative expressing a series of dynamic events. Here, the prefixed verb is associated with a dynamic event with internal phases. This includes initiation (the initiator is unexpressed but clear from the context), but the focus is on the elaboration phase: *ge-dii* expresses a resultant state, which implies an inchoative phase.

Example (14) demonstrates another resultative serialization. Here the alternation applies not to a motion or posture verb, but to *awila′* ‘fill’. In elicitation, the speaker described the meaning difference as a matter of degree; that is, the meaning is neutral ‘fill’ when zero-marked and completive – ‘completely full’ – with an /e/-series prefix. As described above, completive aspect is associated with the end phase of an event, which also implies a greater degree of affectedness of the indexed argument.

(14)

a.

**Table:** 

*ili*	*me*	*bak*	*ite*	*awila′*
water	take	tub	fill	fill
‘(I) fill the tub with water.’				
(Saad fieldnotes, elicited)

b.

**Table:** 

*na*	*ili*	*me*	*bak*	*ite*	*ye-awila′*
1sg.agt	water	take	tub	fill	3./e/-fill
‘I fill the tub with water (completely full).’					
(Saad fieldnotes, elicited)

In (15), *silang* ‘descend’ alternates between a zero-marked variant with habitual reading in (15a), reflected in the translation ‘used to go down’, and a single punctual event denoted by the prefixed variant in (15b). In a habitual reading, the whole event from initiation to completion is relevant since it repeats in its entirety. This contrasts with the prefixed variant, which denotes a single punctual event with internal phases, of which the elaboration phase is in focus: what is important is that the woman has descended, resulting in her being in the hole. This pair of utterances – the only clear example involving habituality identified so far in the corpus – might seem contrary to the previous description of the /e/-series as indicating long duration; however, as mentioned, elaboration phases have telic and atelic characteristics due to including both inchoative and stative phases.

(15)

a.

**Table:** 

*anmante*	*ingkoo*	*sukuu=bo*	*akmi*	*mane*	*mibo*	*ga*	*wo-oi*	*silang=bee*
but.then	earlier	hole=rel	here	village	in=rel	3.agt	3./o/-towards	descend=also
‘but then earlier there was a hole in the village here that she used to go down into …’								
[kamang_aribei]

b.

**Table:** 

*male*	*saak=a*	*ilai-sa*	*yaa*	*me*	*sukuu*	*wo-oi*	*ge-silang=a*
female	old=spec	look-cmp	go	come	hole	3./o/-towards	3./e/-descend=spec
‘[she lay there hiding and] saw the old women go and descend into a hole’							
[kamang_aribei]

As well as intransitive verbs, several alternations involve transitive verbal bases, such as *baila* ‘buy’, *balkei* ‘sell’, and *beta* ‘bump into’. The last-mentioned is a naturally reciprocal event associated with middle semantics (Kemmer 1993: 102–108). The prefix on *beta* ‘bump into’ indicates greater affectedness of P: in elicitation, a speaker explained that unprefixed *beta* means ‘bump into’ or ‘barge’, while the prefixed version means ‘push over’. This follows from greater focus on the elaboration phase of the event, which features P. The corpus yields one alternation pair, shown in (16), which describe the same stimulus (Fedden et al. 2010b), in which man A bumps into man B, causing the latter to step backward. The two speakers frame the event differently: in (16a), man A merely bumps an arm, whereas in (16b), *beta* is prefixed when it indexes man B, who is more affected by the push than an arm.

(16)

a.

**Table:** 

*lami*	*saak*	*nok*	*sue-h=bo*	*ga-tang=a*	*beta*
male	old	one	arrive.ipfv-purp=lnk	3./a/-hand.arm=spec	bump.into
‘An old man comes and hits his arm.’					
(Schapper fieldnotes, elicited)

b.

**Table:** 

*lami*	*saak*	*nok*	*sue*	*ge-nok*	*ge-beta*
male	old	one	arrive.ipfv	3./e/-friend	3./e/-bump.into
‘An old man comes and pushes away his friend.’					
(Schapper fieldnotes, elicited)

The difference in meaning between prefixed and zero-marked *baila* ‘buy’ and *balkei* ‘sell’ is unclear. There are two possible interpretations, both of which fit an elaboration-focus analysis. First, the prefixed variant in (17b) may indicate greater focus on P than A. Indeed, the A argument is not mentioned when the prefix is present, while in the zero-marked variant in (17a), the speaker emphasizes the agentivity of A by using the agentive pronoun.11Agentive pronouns encode S and A, while “basic” pronouns encode any role; what governs the choice between agentive and basic pronouns remains unclear (see Schapper 2014: 315–316).

(17)

a.

**Table:** 

*ga*	*nuaanana*	*baila*	*lai-si=bo*
3.agt	possessions	buy	finish-ipfv=lnk
‘they finished/were finishing buying the things …’			
[kamang_aribei]

b.

**Table:** 

*nuaanana*	*ge-baila*	*lai*	*ge-balkei*	*lai-sa*
possessions	3./e/-buy	finish	3./e/-sell	finish-cmp
‘when (they) had bought and sold their things …’				
[kamang_aribei]

The second analysis of (17) is a more telic interpretation of the prefixed verb: a resultative sense in which (all) the goods have entered into the resultant state of ‘bought’ or a completive sense in which the purchase is complete (compared to ‘being in the process of buying’). The latter interpretation is supported by the co-occurrence of the imperfective suffix *-si* with unprefixed *baila* in (17a).

## 4 Discussion and conclusion

Previous description of DAI with /e/-series prefixes stated that the /e/-series indexes more-patientive S arguments of motion and posture verbs. This paper extends this description in three ways: (i) not all motion verbs alternate, and a heterogeneous group of other verbal bases does alternate, potentially including emotion/cognition verbs; (ii) both S and P arguments can be indexed; and (iii) the alternation is better characterized in terms of elaboration focus. The elaboration phases of an event are the become and state phases, and entail a cluster of properties including features of a prototypical P argument (greater affectedness, low volitionality), completive aspect, resultativity, and features of middles. These properties – evoked individually or in combination – were demonstrated by several alternations, alongside an additional meaning difference whereby the prefixed verb denoted a punctual event and the zero-marked verb had a habitual reading.

Elaboration constructions such as resultatives and middles have a cross-linguistic tendency towards lexical restriction. This was already found for DAI in Kamang in general (Fedden et al. 2013, 2014; Walker et al. 2023), and was demonstrated here for /e/-series DAI: the number of verbal bases that alternate is quite low, not all verbal bases in a particular semantic class can alternate, and alternation often leads to lexicalized meaning differences.

The type of DAI alternation in which elaboration phases receive special marking was termed an initiation-elaboration split, exemplified by Pilagá in Section 2 and shown in the first line of (18). Kamang’s /e/-series alternation differs from an initiation-elaboration split proper in that it is asymmetrical in two ways. First, it is asymmetrical in the formal sense of alternating between a zero and a non-zero marker, rather than between two different affixes (see Witzlack-Makarevich and Seržant 2018: 23–25). Since Kamang does not index A arguments, the formal asymmetry reflects the fact that indexing the initiator is not an option to signal initiation focus. Second, it is asymmetrical because a zero-marked verb expresses a holistic view of the event, either with all three phases, or with no internal phases. This is schematized in (18).

(18)

**Table:** 

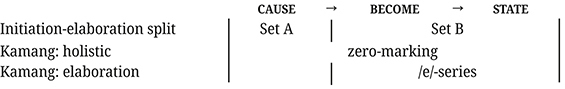

The ambiguity of zero-marking is unsurprising, given that zero in Kamang alternates not only with the /e/-series, but also with other prefix paradigms (as well as being the only option available for some verbal bases). That is, this is not a binary alternation, but a multidimensional one in which the default meanings of unprefixed verbs can be manipulated. Where other languages use voice or highly developed aspect systems, Kamang speakers have differential /e/-series indexing alongside predominantly non-obligatory aspect marking and resultative serialization to manipulate the semantics of a predicate. Though highly dependent on individual verbal bases, in general an /e/-series index will shift focus to the elaboration phase of an event. Further research – utilizing additional methodologies such as controlled experiments – is needed to confirm the description advanced here, which is based on the qualitative analysis of a small number of tokens. This should also test the acceptability of /e/-series prefixes on additional verbal bases and the degree of optionality on the verbal bases already identified, investigate the compatibility of DAI with aspectual marking, and compare the /e/-series alternation to the /o/-series alternation, which has also been described as indicating greater affectedness (Schapper 2014: 325).
